# Interactions between a population of fallow deer (*Dama dama*), humans and crops in a managed composite temperate landscape in southern Sweden: Conflict or opportunity?

**DOI:** 10.1371/journal.pone.0215594

**Published:** 2019-04-23

**Authors:** Lorenzo Menichetti, Laura Touzot, Katarina Elofsson, Riitta Hyvönen, Thomas Kätterer, Petter Kjellander

**Affiliations:** 1 Department of Ecology, Swedish University of Agricultural Sciences, Uppsala, Sweden; 2 Laboratoire de Biométrie et Biologie Evolutive, Université Claude Bernard Lyon, Lyon, France; 3 Department of Economics, Uppsala, Sweden; 4 Grimsö Wildlife Research Station, Department of Ecology, Swedish University of Agricultural Sciences, Riddarhyttan, Sweden; CONICET - Universidad Nacional de Tucumán, ARGENTINA

## Abstract

Landscapes composed of agricultural land mixed with forest are desirable since they provide a wide range of diversified ecosystem services, unlike specialized agricultural landscapes, but that creates a trade-off between these land uses since wildlife usually feed on crops and reduce yields. In Nordic countries, where human population density is low and game hunting can be a viable economic alternative, mixed landscape systems are particularly interesting. To evaluate the economic sustainability of such systems we need to quantify wildlife damage to crops. One important species, being popular among Swedish hunters and therefore economically valuable, is fallow deer (*Dama dama*). Our objective was to evaluate the economic sustainability of mixed landscape systems including cultivated fields and commercial hunting of fallow deer. We studied the effects of excluding fallow deer by using 86 exclosures and adjacent plots in winter wheat and oat fields in south-west Sweden. We analyzed yield losses and interactions between spatial and temporal grazing patterns, anthropogenic landscape features, and topological characteristics of the landscape. We found that animals avoided exposed spots, irrespective of distance from human activity. We also found a seasonal grazing pattern related to the different growing periods of winter wheat (more grazed, emerging in autumn) and spring oat (less grazed, emerging in spring). We then compared the costs of crop damage against the commercial value of fallow deer hunting. The damage amounted to 375 ±196 € ha^-1^ for wheat and 152 ±138 € ha^-1^ for oat, corresponding to a total cost per animal of 82.7 ±81.0 €, while each animal had an estimated market value of approximately 100 €. Therefore the value of fallow deer presence compensated for the associated cost of crop damage. Profit could be further improved in this case by adopting additional management strategies. In general our study confirmed the economic feasibility of this particular mixed land management.

## Introduction

Global demand for food is expected to increase substantially in the future. However, food production is often in conflict with other land-based ecosystem services relating to wildlife, since the objective of food production implies the minimization of ungulate density in order to avoid damage to crop. But wildlife can provide many other ecosystem services such as higher spatial and species diversity, carbon sequestration potential [1–3], erosion control [4] and recreational and cultural services [5,6] which make a high wildlife density desirable. The population density of wild ungulates has been increasing in Europe in recent decades [7]. Many wild ungulates have opportunistic feeding behaviors and feed on crops [8,9], so their impact on economic profit is generally considered negative [3]. Compensation for wildlife damage to crops is the second largest class of agricultural compensation of any kind world-wide, after livestock damage caused by carnivores. It represents 35% of the total global amount of compensation to agriculture (which has been on average approximately 8 million € per year over the past five decades), even though its effectiveness in mitigating human-wildlife conflicts is still not well proven [10]. Moreover, the reduction in landscape complexity caused by agricultural intensification could threaten the resilience of agricultural environments [11], since landscape heterogeneity has been positively related to ecological processes such as pollination [12]. Landscape heterogeneity is also correlated directly with perceived landscape recreational value [13] and with recreational [14] and cultural [15] ecosystem services. In temperate environments, increasing landscape heterogeneity means finding a practical solution to manage landscapes incorporating forests and cropland, which can be achieved through increasing the economic sustainability of such systems.

A particularly interesting component in mixed landscapes is ungulate populations managed for recreational hunting, since the animals in this case have a monetary value. In landscapes composed of cropland mixed with forest, this value could possibly compensate for crop yield losses caused by grazing, making the system a viable option for landowners. In Sweden, fallow deer (*Dama dama*) are particularly relevant in this context. Fallow deer is a non-native species first introduced to Sweden in the mid-16^th^ century [16] and now present in all southern parts of the country [17]. It thrives in mixed environments and is often intensively managed for its hunting value. The estimated number of wild fallow deer harvested in Sweden is > 40,000 per year (Swedish Hunters Association; https://rapport.viltdata.se/). This is around half the number of kills per year on a national scale of native species such as moose (*Alces alces*) and roe deer (*Capreolus capreolus*), and recently re-introduced wild species such as wild boar (*Sus scrofa*). Fallow deer exhibit opportunistic grazer feeding behavior and, compared with other Nordic cervids, feed more often on agricultural crops [18]. While low animal density is desirable for limiting the damage to crops, high population density of a popular game species might have high recreational and monetary value, and these two opposite objectives define a trade-off between animal density (hunting value) and crop production (yield). High fallow deer population density in agricultural areas potentially results in reduced production levels, through animals feeding on the crop at different stages of plant growth. However, unbiased estimates of crop losses in areas with known ungulate densities are rare, often inconsistent, and often limited to grey literature [19–21] or to self-evaluation qualitative surveys of farmers reported in non-peer-reviewed sources [22]. This lack of data is particularly severe at the field scale with spatial characterization, despite that interactions between human interventions and herbivore diet are well known [23,24]. Available studies are usually on small areas [1,19,20]. Their results are difficult to extrapolate to new environments and population densities and clearly, there are no previous studies from Northern Europe qualifying the impact of a large herbivores on crop production and associated costs or benefits. Given the low human population density and the diffusion of hunting as a sport, Nordic countries are among the best candidats for mixed landscape management including game hunting. A few recent publications on crop damage caused by ungulates cover other species, e.g.,[25] consider a mixture of wildlife species including white-tailed deer, but not fallow deer. Other previous studies have focused on distance from anthropogenic landscape features, neglecting more complex topological interactions [26]. Accurate quantification in monetary terms of deer damage to crops and a better understanding of the spatial patterns and drivers of such damage could assist in the development of management strategies and mitigate unavoidable conflicts arising in landscapes with mixed land use.

The aims of this study were to (1) identify possible spatial patterns of fallow deer grazing in croplands and any spatial interactions between landscape features and grazing intensity (crop losses), (2) quantify any crop harvest losses due to grazing by free-ranging fallow deer, (3) assess the economic sustainability of a diversified wildlife/crop landscape, and (4) suggest improvements to management strategies.

First, we estimated the spatial distribution of crop losses in exclosures and in adjacent plots in oat (*Avena sativa*) and winter wheat (*Triticum aestivum*) fields on an unfenced estate in south-western Sweden managed for crop and forest production together with commercial recreational hunting. We then evaluated the relationship between this distribution and distance from human activity hotspots, forests, and topological features such as interactions of visibility and openness in this particular landscape. We utilized the data obtained to assess the economic feasibility of combined wildlife-crop-forest management at the study site and to formulate suggestions for improvements.

The underlying assumption was that combined wildlife-crop-forest landscape management at the study site represents a potentially viable model for building a diverse temperate-environment landscape that is still productive and economically viable. Good economic sustainability could incentivize creation of similar settings by other large landowners.

## Materials and methods

### Study area

The study site was located on the Koberg estate in south-west Sweden (58°08’50”N, 12°24’40”E). Permission to execute the study was granted by the Koberg estate (land owner). Part of the estate is dedicated to commercial production of cereals. The density of the fallow deer population in the area is high compared to average densities of large ungulates over Europe(e.g. [27]), estimated as 15 individuals per km^2^, while the density of other large herbivores is negligible.

**Fig 1:**
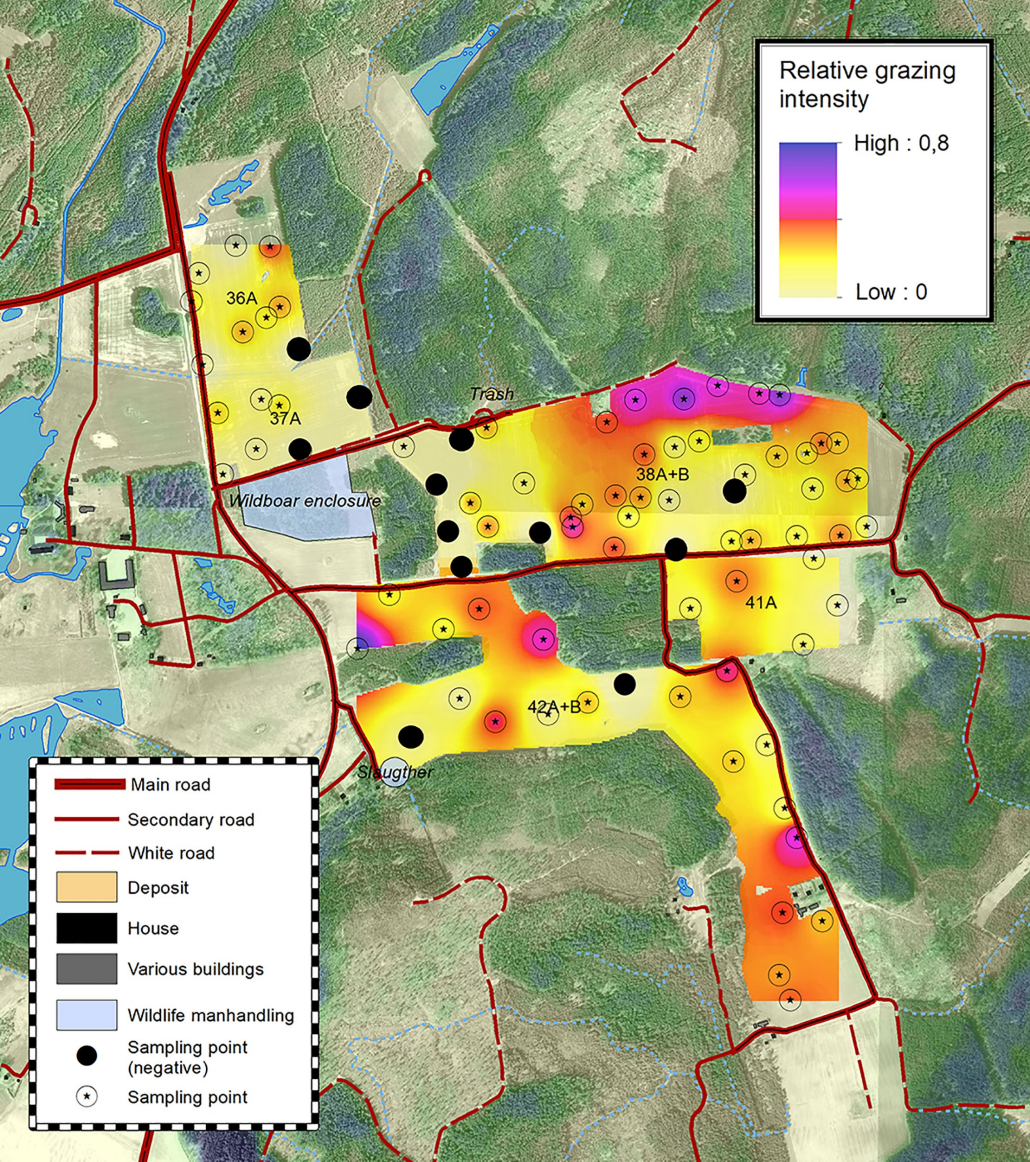
The browsing ratio in the studied fields. Fields 36A, 37A and 38A and B were planted with oat, while fields 42A and B where planted with winter wheat. The points identified by a solid black dot are the negative values, where crop inside the deer exclosures had lower biomass than outside. Grazing data are plotted after IDW interpolation, and negative pixels are set to zero for readability purposes. False colors in the ortophotograph, from light green to blue, express the height of the tree coverage.

Most (80%) of the Koberg estate is covered by forest dominated by Scots pine (*Pinus sylvestris*) and Norway spruce (*Picea abies*), but highly fragmented by agricultural fields. Forest stands in the study area are cut every 45–80 years, depending on tree species and local stand conditions. Since 2004, a wildlife fence along the main road to prevent deer-vehicle collisions has split the estate into two parts. In the northern part managed for crop production and forestry (where this study was based), wild animal density is limited by programmed hunting. The southern part of the estate is managed exclusively for forestry and commercial recreational game hunting (where customers pay for the right to hunt on the property) and higher wild animal density is permitted in that area. The specific location where our study took place, north of the main road (Fig 1), comprises 9,300 ha, of which approximately 3.9% were under cultivation at the time of the study (3.0% winter wheat, 0.9% oat). Deer feeding sites are located on the whole property, but are used only during winter and only to meet the basic maintenance requirements of the deer population when food is scarce. Despite these feeding sites, animals still face starvation during the winter. Food availability for ungulates in temperate environments has been found to be strongly inversely correlated to snow cover depth [28] and therefore varies widely both within and between years. The climate at the study site is warm temperate, classified as temperate oceanic climate, Cfb, in the Köppen-Geiger classification system [29]. Mean monthly temperature is below 0°C for two or three months per year (January-February and parts of December and March), with a mild monthly summer temperature of above 10°C from May to September. Mean temperature over the whole year is 8.4°C. Precipitation is evenly distributed throughout the year, with an average of 686 mm per year.

### Study species

Fallow deer is one of the most widely distributed species of deer in the world[17]. However, relatively little research has been done on free-ranging fallow deer in Northern Europe and Scandinavia [16,30]. The species is hunted during a regulated hunting season, as are all other native game species. Fallow deer originates from the Middle East and was introduced to Sweden by the nobility as game for hunting in parks around castles and estates [17]and later more actively managed in fenced areas (deer parks). From these areas, individuals were released or escaped and started forming feral populations, and the species is now well distributed in the form of scattered occurrences over the southern third of Sweden [17,30,31]. Although the fallow deer population has increased dramatically (more than 100-fold) in the past century and particularly in the past two decades, it is still numerically smaller than the population of native moose and roe deer, but much more abundant than the native red deer [31]. Fallow deer has high economic importance because of its value as game and for commercial hunting. The reported national annual harvest of fallow deer in the wild has increased from 1,000 animals in 1939, the first year with official statistics, to >40,000 in 2017. At the local scale (Västra Götaland county), annual harvest rates have varied between 2,000 and 4,000 animals during the past seven years (https://rapport.viltdata.se/statistik/). With an increasing population, the need for good management strategies increases.

### Experimental methods

The experimental treatment applied in the present study consisted of 172 plots (86 pairs) half of which were equipped with exclusion cages to prevent deer feeding. In total, 59 cages were installed in oat fields and 27 in winter wheat, uniformly covering all the field space assigned to the study. The cages were placed in five official cultivation blocks registered with the local land authority (36A, 37A, and 38 A+B for oat; 41 and 42 A+B for wheat). Controls and cages were installed in pairs that were distributed over the study fields in order to fill the area with approximately the same density. Each pair was separated by approximately 10–25 m. Each cage was approximately 1.20 m high and covered a square measuring 1 m × 1 m and was constructed as a wooden frame supporting metal netting with mesh size 10 cm × 5 cm. The cages were secured in the ground with U-shaped pegs and then georeferenced with a hand-held GPS unit (Garmin CSx and later models). The cages were put in place on the day after sowing, on August 26, 2013 for winter wheat and on April 23, 2014 for oat. Note that winter wheat is sown in fall and emerges before winter, and is thus much earlier than oat. Winter wheat can germinate at temperatures as low as 4°C and the plants enter a vegetative state (adapted to the cold) throughout the winter, a process called vernalization.

The crops were harvested manually (using scissors) at maturity, which for winter wheat was on August 4, 2014 and for oat on August 12, 2014, both inside the cages and in the control plot of the same size. The harvested material was collected in paper bags and dried for 3–7 days at 60°C. The dried samples were first weighed as total biomass, with straw and grain, and then only as the cleaned grains. A subsample of bags was carefully screened for weeds, which accounted for 0.7% of the biomass in the oat crop and 0.3% in wheat. These values were used for correcting all the biomass values and the effect of the deer exclusion treatment was considered as the difference in biomass between inside and outside the cages (expressed per hectare). In some cases the difference was negative. In the calculations, we decided to interpret all negative values as a null effect of the treatment, but later we also briefly discuss the negative results and offer a potential explanation.

The fallow deer density in the study area was estimated in April with a Distance Sampling procedure [32] by walking 40 transects, each 1 km long, for 2–3 times over a period of 3 weeks. The transects cover approximately the whole study area.

### Geographic data sources

Data on the location of roads and buildings and a digital elevation model (DEM) of the terrain were obtained from the Swedish National Land Survey. Laser Imaging Detection and Ranging (LIDAR) data for 2011 and 2012 were also obtained from the Swedish National Land Survey, together with ortophotographs from the same sources taken in 2014 for the lower part of the study area (mostly the wheat field) and in 2012 for the upper part (mostly the oat field). There was substantial agreement between all sources, in particular tree height maps calculated from the LIDAR data and the ortophotographs (Fig 1). Two local features (a wild boar enclosure and a slaughterhouse) were mapped during field surveys.

### Data analysis

Data analysis was performed in ArcGIS (version 10.4.1, ESRI, Redlands, California, USA) and R [33]. Tree height was estimated by subtracting the DEM layer data from the LIDAR layer data. The main geographic analyses performed were proximity and topological analyses.

We calculated the distance of each sampling point from forest boundaries (determined by applying a threshold of 1.5 m height on the tree height raster previously calculated) and from features that could be expected to affect animal behavior, such as houses, roads, the wild boar enclosure, and the slaughterhouse. Using the three-dimensional LIDAR data, we also ran topological analyses (3D Analyst toolbox in ArcGIS). The inclusion of visibility when analyzing the effect of roads on wildlife is suggested to be important for understanding landscape-wildlife interactions [26]. We therefore calculated a “visibility from road” raster for the study fields, where pixel value expressed the probability of being seen inside that pixel by an observer passing by on nearby roads. Each sampling point (cage location) was associated in this way to the corresponding visibility value. The local forest has a dense understory, and therefore the visibility parameter was a measure of how the point was exposed to or shielded from roads by vegetation. We also calculated the openness as a “sky footprint” polygon for each observation point, by first calculating the skyline based on the LIDAR and then the skyline barriers (Skyline and Skyline Barrier tools[34], which represent the boundaries where the sky meets any surface feature observable from the perspective of each observation point. We then calculated the area defined by the skyline barriers. This value represents the portion of sky visible from each point, and therefore it relates to the generic “openness” of a certain point regardless of road interactions, which has been found to affect the behavior of some deer species [35].

We tested the impact of each variable in explaining the observed variance in grazing intensity by linear multiple regression using the equation:

$$Grazing∼f\left(dist_{f}+dist_{r}+dist_{t}+visib+open+biom\right)$$ Eq 1

where *Grazing* is grazing pressure (the difference between biomass in each exclosure and in the grazed surroundings, normalized between 0 and 1), *dist*_*x*_ is distance from man-made features (*f*), roads (*r*), or forest edges (*t*), *visib* is visibility, *open* is openness of the point, and *biom* is absolute amount of biomass produced at each point. The relative importance of the components and the associated uncertainty were evaluated by bootstrapping (R package *relaimpo*; Grömping, 2006). The metric chosen was *lmg*, as recommended by the package author, which is equivalent to the hierarchical partitioning approach [36] and decomposes the overall R^2^ of the regression into relative components that add up to 1. These were multiplied by the R^2^ itself to obtain an absolute measure of overall variance. Values calculated per hectare were calculated by area average (geometric mean) over the whole cultivated surface. All data derived from the sampling points were interpolated into a raster with inverse distance weighting (IDW). Values were considered statistically significant at p<0.05 unless otherwise specified.

### Economic analysis

The overall cost of grazing damage was calculated as loss of revenue for the landowner. The costs of plowing, sowing, harvesting, and transport were assumed to be unaffected. This is a simplification, as the costs of combine harvesting, drying, and transport could be reduced due to the lower yield. Thus, our cost estimates correspond to the reduction in the landowner’s short-run contribution margin, based on revenues from sales and costs for variable inputs except labor costs. We used average national cereal prices from the Swedish Board of Agriculture for 2014 for calculating the impact on landowner revenues in absolute terms. We used the farm business calculation program Agriwise [37] to calculate the relative impact on landowner profits. All amounts are given in Euro (€), based on a conversion rate of 9.0968 Swedish Krona to the Euro, which was the rate at the time of the study. The damage (in Mg ha^-1^) and cost per animal unit were measured on a fully farmed area because of our experimental design (the area covered by the cages extended over farmed fields). The results are expressed as feeding effect per animal (cost per hectare divided by animal density) with respect to a hypothetical reference area with 1% of the land being used for winter wheat or oat, so divided by 100. This value is reported in order to allow application of the results to areas with different crop densities. For our subsequent calculations, we then rescaled these values based on the area effectively farmed on the Koberg estate, which is 3% of the total estate area for wheat and 0.9% for oat. The estimated cost per animal of the damage was calculated as the feeding effect per animal multiplied by the price of each specific crop.

The estimated cost per animal was then compared against the value of having fallow deer on the land. Since there were no direct empirical data available on this value, we roughly estimated it based on the value of harvested deer. For the property in question, the average net revenue of harvested fallow deer, based on net revenues to the landowner from commercial hunting events plus meat sales, is approximately 500 € per fallow deer shot [38]. Another study estimating fallow deer hunting value [39] reports that hunters’ willingness to pay for fallow deer is about 300 € per deer shot. The lower value in the latter study is because it applies to non-commercial hunting, which is less relevant in the present case.

## Results

### Spatial pattern of fallow deer grazing

**Fig 2:**
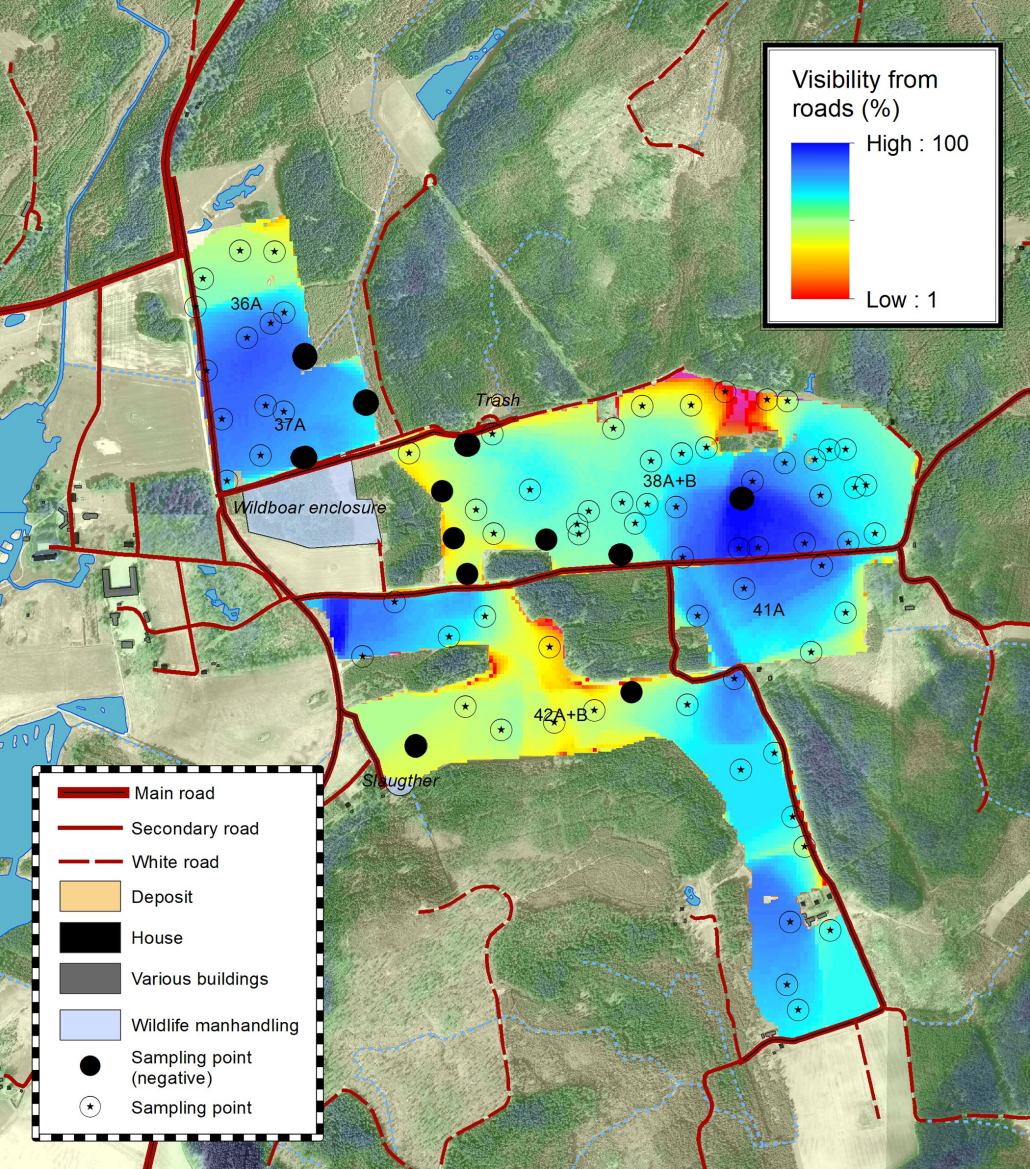
The visibility from roads in the studied fields. Fields 36A, 37A and 38A and B were planted with oat, while fields 42A and B where planted with winter wheat. The points selected in bold light blue are the negative values, where crop inside the deer exclosures had lower biomass than outside. False colors in the ortophotograph, from light green to blue, express the height of the tree coverage.

**Fig 3:**
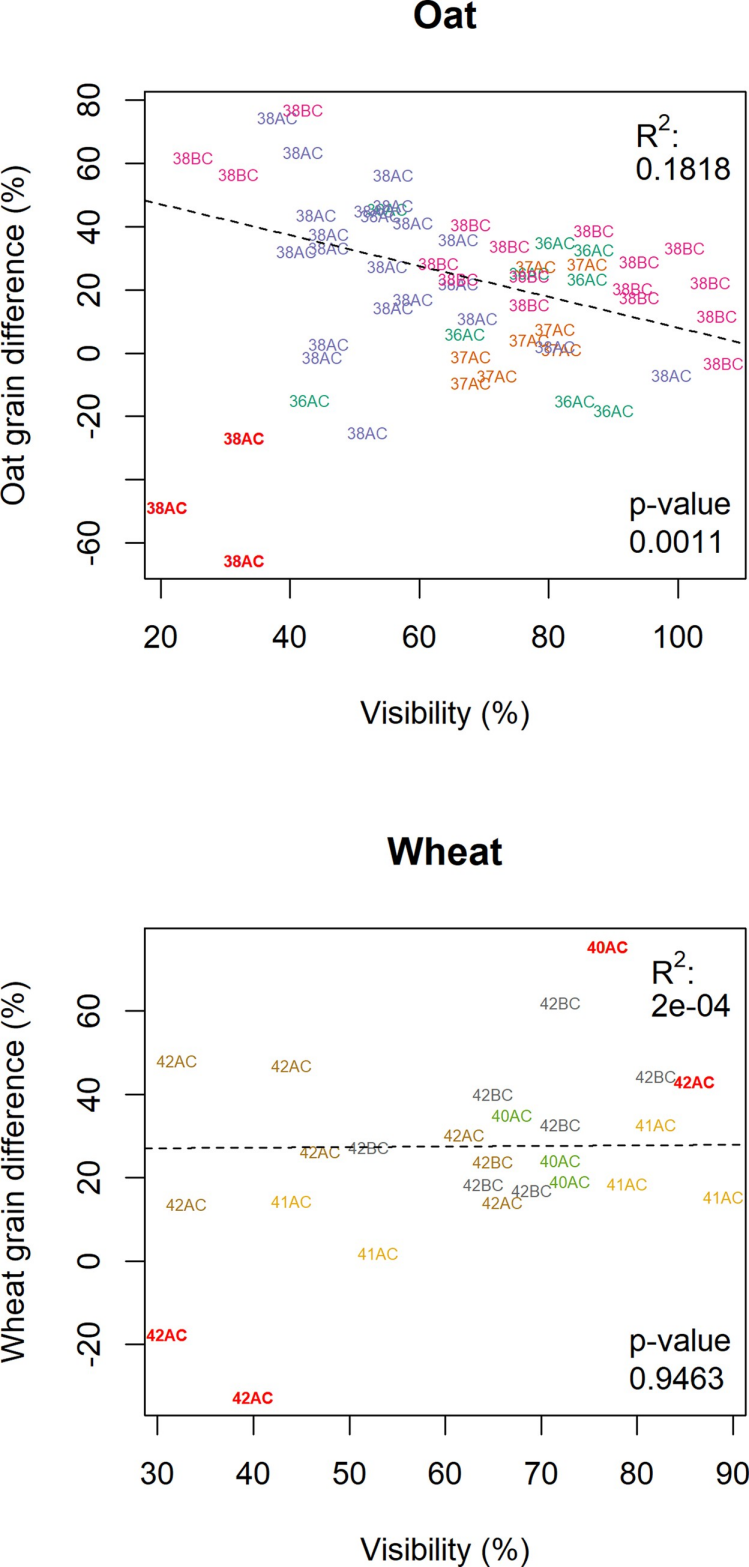
Correlation between grazing intensity and the visibility of the sampling spot for the studied crops. Each alphanumerical code corresponds to fields in Figs *1* and 2. Red bold character indicates the identified outliers.

**Fig 4:**
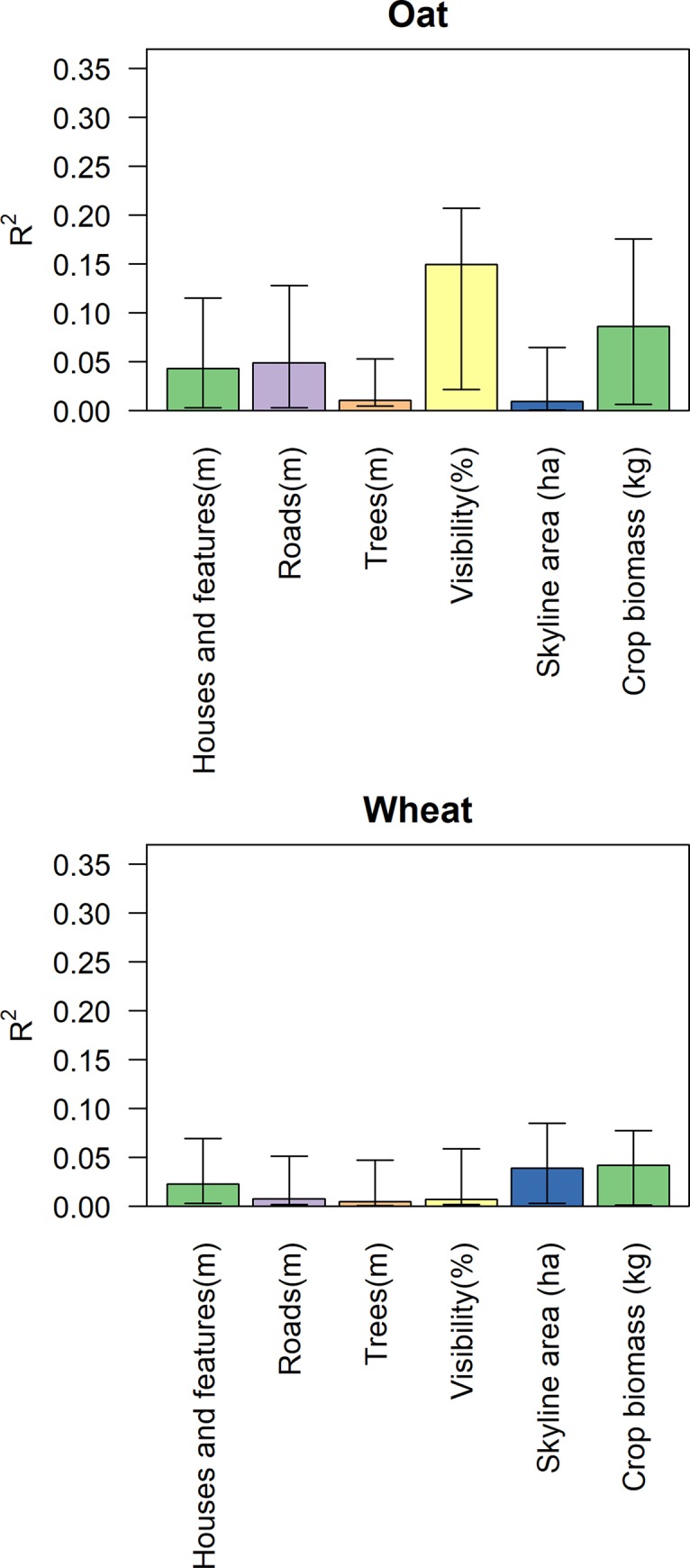
The decomposition of the importance of the multiple linear regression. Correlation is expressed here as absolute R^2^ values. Bars represent the explained part of the grazing pressure (as described by Eq 1) by each parameter expressed in relative weight, lines represent the error of the estimate.

The regression model described by Eq 1 explained 35% of the variance for oat and 12% of the variance for wheat. A greater proportion of variance was explained by the topological analyses in the case of oat. In particular, visibility from road (Figs 2 and 3) explained 15% of total R^2^ for grazing intensity in the oat fields (p = 0.0011), but virtually none of total R^2^ for grazing intensity in the wheat fields. Distance from roads, other man-made features, and forest edges each explained at most 4.9% of total R^2^ of the observed spatial patterns for oat and 2.2% for wheat (see Table 1 for the relative importance weighting of each parameter and Fig 4 for decomposition of R^2^).

**Table 1:** Relative importance of the variables tested in Eq 1 in explaining the observed variation in grazing pressure (mean value, lower and upper 95% confidence interval in brackets). These values refer to the part of the overall R^2^ explained by each component.

	Oat	Wheat
Houses and features (m)	0.12(0.01–0.38)	0.18(0.03–0.55)
Roads (m)	0.14(0.01–0.44)	0.06(0.02–0.39)
Trees (m)	0.03(0.01–0.17)	0.04(0.01–0.4)
Visibility (%)	0.43(0.06–0.69)	0.06(0.02–0.48)
Skyline area (ha)	0.03(0–0.22)	0.32(0.02–0.68)
Crop biomass (kg)	0.25(0.02–0.59)	0.34(0.01–0.61)

We observed a defined spatial grazing pattern in the oat fields, but not in the wheat fields (Fig 1). Grain and total plant biomass were strongly correlated (R^2^ = 0.952, p < 0.0001). We also obtained a few negative values for grazing intensity (where negative value means that the plants in the caged plots, protected from deer feeding, yielded less total biomass than the plants subjected to deer feeding). The number of plots in which there was less total biomass inside the exclosure than outside was 13 and 2 for oat and wheat, respectively. Very similar results were obtained for grain biomass estimates, with 13 and 3 negative plots for oat and wheat, respectively (Figs 1 and 2). Most of the recorded negative values for oat were clustered just east of a small wood close to the wild boar enclosure (Fig 1). All the negative values except one were recorded close to the field borders and close to a forest patch that could act as potential shelter.

A weak but significant relationship was found between grazing intensity and absolute biomass in the caged plots. In particular, the multiple linear regression model explained a significant part of the total variance (R^2^ = 0.349, p = 0.0013) in removed oat grain biomass, while wheat grain yield relationship was non-significant (p > 0.8 in all cases).

### Overall effects of fallow deer grazing on grain yield

The absolute grain loss recorded in winter wheat was (all values are reported as mean ±SD) 1.89 ±0.62 and 2.44 ±1.3 Mg ha^-1^ for blocks 41A and 42A+B, respectively (overall mean 2.33 ±1.22 Mg ha^-1^, differences between the blocks were not significant in an ANOVA loss ~ crop × block). Losses relative to the control plot were 22.45 ±28.4% for grain biomass and 26.3 ±23.3% for total winter wheat biomass.

The estimated grain loss recorded in oat was 0.80 ±0.58, 0.38 ±0.43, and 1.48 ±1.16 Mg ha^-1^ in blocks 36A, 37A, and 38A+B, respectively (overall mean 1.21 ±1.1 Mg ha^-1^, differences between the blocks were not significant in an ANOVA loss ~ crop × block). Losses relative to the control plot were 19.9 ±27.7% for grain biomass and 18.9 ±27.4% for total oat biomass.

Fallow deer density in the grazing area was found to be approximately 0.15 ±0.04 animals ha^-1^. The feeding effect per animal amounted to 0.151 ±0.079 Mg ha^-1^ for wheat and 0.079 ±0.071 Mg ha^-1^ for oat, while the specific grain biomass loss 0.460 ±0.241 Mg ha^-1^ for wheat and 0.069±0.063 Mg ha^-1^ for oat when accounting for the areal coverage of wheat (3%) and oat (0.9%).

### Monetary value of fallow deer grazing

Using 2014 prices for wheat and oat (0.160 € kg^-1^ and 0.125 € kg^-1^, respectively), each deer caused damage corresponding to 74 ±39 € for wheat and 9 ±8 € for oat, i.e., a total cost per animal of 83 ±81 €.

Losses per hectare cropland in 2014 amounted to 374 ±196 € ha^-1^ for wheat and 152 ±1380 € ha^-1^ for oat. Scaling these values up to the whole property, which in 2014 grew 143 ha of wheat and 82 ha of oat, resulted in total damage due to deer feeding of 53,692 € for wheat and 12,475 € for oat, giving a total loss of more than 66,000 €. This corresponds to a reduction in the short-run contribution margin of 27.7% for winter wheat and 30.2% for oat, giving a reduction in total short-run profits from the two crops of 28.3%.

In the study area, on average 20% of the deer population was harvested each year between 2007 and 2013. Given the estimates of the value of harvesting deer, mentioned above, this suggests that the value of having one fallow deer on the land is approximately 100 €. Hence, the average value of having deer on the land in this case slightly exceeded the average cost of crop damage (83 ±81 € per deer).

## Discussion

We found that animals avoided exposed spots, irrespective of distance from human activity (aim 1). We also found a seasonal grazing pattern related to the different growing periods of winter wheat (more grazed) and spring oat (less grazed). In monetary terms, the value of fallow deer presence compensated for the associated cost of crop damage (aim 2). Below we will discuss these results and its implications in more detail.

### Grazing patterns and anthropogenic landscape influence in oat and wheat crops

The absence of a clear spatial feeding pattern in wheat fields and of a strong pattern in oat fields could be explained by the different growing seasons of the two crops. Winter wheat is sown in early fall and nutritious shoots emerge quickly and are available throughout the winter if the snow cover is shallow or absent (as was often the case in 2014 during this study), when natural forage is sparse and of low quality. On the other hand, oat are sown late in spring and the plants develop during the spring flush of other highly nutritious natural forage. Thus, while harvesting was at the end of summer for both crops, wheat was a less attractive food than oat after emergence of the oat plants and definitely after the stage of full grain development [40]. Therefore, the agronomic and phenological cycles of winter wheat expose it to deer feeding in winter and fall, while oat become a viable source of feed late in spring, when many other feed sources are available.

Fallow deer tend to prefer a mixed diet [41,42], and during the Scandinavian winters they are likely to struggle to find enough natural high-quality forage [43,44]. Before the spring vegetation flush but when wheat shoots are already available, in the absence of natural food sources and facing a negative energy balance, deer could be forced to take greater risks. During winter the animals would therefore feed in more exposed parts of fields thus ignoring much more topographical features, while in the summer an eventual deterrent effect of topographical features would emerge better given the wider range of feeding choices available to the animals that would then forage on oat only whenever some kind of protection is offered. This might explain why there were no significant effects of topographical features or even palatability on wheat, while there were clear spatial effects on oat fields, marking also a seasonal pattern in grazing. Another possibility to explain the absence of spatial pattern in feeding on wheat could be explained by the low visibility in winter due to the few hours of daylight at these latitudes and thus a possibility to feed in the protection of darkness, in contrast to the summer when there are very few hours of protection during night and thus making physical protection much more important.

Results compatible with a seasonal pattern in the risk behavior of animals in relation to food availability have been reported previously for fallow deer by Bruno and Apollonio [41]. Although their study was conducted in a different landscape, in a Mediterranean ecosystem, they found that the reliance of fallow deer on trees and shrubs decreased from spring to autumn, when the deer grazed more nutritious graminoids. Similar behavior has been reported previously for alpine red deer [45].

In general, distance from roads, houses, or other anthropogenic features, or distance from the forest, did not seem to influence fallow deer feeding patterns. Moreover, the openness of the area (expressed as sky footprint) did not seem to have a strong influence on deer grazing patterns. The spatial feeding pressure on oat seemed instead to be driven by visibility from roads (Fig 2), which contributed to about half of the whole predictive power of the model (Fig 4). Similar behaviors have been observed for other deer species such as red deer [46].

The grazing patter in oat crops suggest that deer behavior could be driven by a relatively detailed risk evaluation, as they seem able to consider that short distance from human activities does not constitute a risk *per se*, but that being visible to humans passing by is a risk. It is indeed the highest risk factor, since deer in the study site are hunted by humans and learn to fear them [47]. The ability to perform such complex risk evaluations, and to modify behavior accordingly, has been observed in other studies of large herbivores such as red deer [48], reindeer [49]. and also fallow deer [50]. In general the spatial pattern of grazing in the spring seemed to be influenced by a combination of field shape and road position, but the observed effect of visibility might be related to some local characteristics or be specific to oat, and its generalization must be considered with caution.

### The economic sustainability of game wildlife in a landscape characterized by cropland mixed with forest

So far, the only available source of estimated wildlife damage to crops in Sweden is a study from 2014 based on a farm survey. Fallow deer were reported by farmers in Västergötland county, where our study area was situated, to be responsible for 9.1% of total harvest losses in oat and 7.5% in wheat [51]. At a national scale, the reported losses due to wildlife are on average rather small, in particular compared with those observed in our study, but with very high variation. These data are based on farmer reports and expected revenues, so they present a high uncertainty compared to the results of an exclosure experiment. The crop harvest losses we recorded relative to the production level inside the exclosure cages was substantially larger, and might suggest that the national estimates underestimate the damage. Given the roughly similar grain production in the unprotected plots and at the national level, we believe that our study area can be considered representative of crops on the national scale. Our data suggest higher losses to wildlife than previously suggested, at least for managed fallow deer populations.

The value of having deer for the purpose of commercial hunting exceeded the costs of the associated crop damage in our study system. This does not mean that the landowner’s best choice is to accept the damage and there may be management choices that could minimize the damage. In general, the crop damage costs were significant enough to justify adoption of management-based measures whenever possible and also to justify adoption of more input-intensive measures (e.g., fencing), although this would need to be considered in relation to the management strategy of the whole property (for example this might eventually require to increase the amount supplementary winter food provided to the deer). The outcome would depend on many local factors such as whether the farmer can actually commercialize fallow deer hunting and sell the meat, whether the person growing crops owns the hunting rights, the local density of game animals, and the type and amount of crops. An increase in the profit margin might therefore be important to generalize the profitability of this kind of management.

For perspective, a possible cost mitigation strategy would be to fence the whole perimeter of the fields (approximately 15 km in the northern part of the Koberg estate) with woven wire, which according to [52]is the most effective type of fence. According to those authors, the investment cost for such wire is 8.2–12.5 € per meter, and it has a lifetime of approximately 30 years and an efficacy of 90%. At a 10% depreciation rate, the annualized investment cost is then 13,114–19,765 € per year, or 20–30% of the recorded economic losses, and the measure would therefore be profitable. Other management strategies, for example aiming at modifying the behavior of the animals with specific hunting strategies intended to produce a fear response [47], minimizing damage through crop management, or attempting to divert animal feeding away from key growing phases for crops by establishing artificial feeding sites far from cultivated fields [53], could also make this type of mixed forest-cropland landscape more profitable in general. Another possibility to minimize deer damage close to the fields would be to increase the visibility in the immediate surroundings by targeted forest management, for example reducing tree density or eliminating the understory, as this would increase the predation risk from hunters [54]. Our data suggest that increased visibility in the surroundings of crop fields could also directly modify animal behavior during the spring. Another possible low-cost management alternative could be applying hunting strategies aimed at increasing the fear response of animals to roads and other features that could exert some sort of protective effect on crops [47].

To summarize, our study supports the overall economic sustainability of a diversified crop-forest-wildlife hunting landscape in south-west Sweden. This land use combination could be a viable option in temperate environments, combining in one landscape multiple ecosystem services such as food production, recreational hunting, and cultural and environmental values of wildlife, particularly if additional preventive measures are employed.

## Supporting information

S1 Dataset core_data.csv. (ZIP)
